# Non-linear association of atherogenic index of plasma with bone mineral density a cross-sectional study

**DOI:** 10.1186/s12944-024-02180-3

**Published:** 2024-06-12

**Authors:** Bo Xu, Guoliang Ma, Liu Yang, Xin Chen, Bo Bian, Bowen Yang, Dian Zhang, Xiaokuan Qin, Liguo Zhu, He Yin, Xu Wei, Minshan Feng

**Affiliations:** 1 Department of Spine, Wangjing Hospital, China Academy of Chinese Medical Sciences, Beijing, 100000 China; 2 Department of Dermatology, Guanganmen Hospital, China Academy of Chinese, Medical Sciences, Beijing, China; 3 Traditional Chinese Medical College, North China University of Science and Technology, Tangshan, Hebei China; 4 Beijing Key Laboratory of Bone Setting Technology of Traditional Chinese Medicine, Beijing, China

**Keywords:** Bone mineral density, Atherogenic index of plasma, Osteoporosis, Cross-sectional study, NHANES

## Abstract

**Introduction:**

Although there has been abundant evidence of the association between dyslipidemia as a single factor and osteoporosis, the non-linear relationship between osteoporosis and the Atherogenic Index of Plasma (AIP) has not yet been thoroughly investigated. This study aimed to investigate the complex relationship between AIP and bone mineral density (BMD) to elucidate their interrelationship.

**Methods:**

An analysis of 2007–2018 National Health and Nutrition Survey (NHANES) data was conducted for this study. The study enrolled 5,019 participants. Logarithmically multiplying triglycerides and high-density lipoprotein cholesterol yields the AIP (base 10). The measured variables consisted of BMD in the total femur (TF), femoral neck (FN), and lumbar spine (LS). The association between AIP and BMD was examined using a range of statistical models, such as weighted multivariable logistic regression, generalized additive model, etc.

**Results:**

It was found that AIP was positively associated with BMD after adjusting for age, gender, race, socioeconomic status, degree of education, income, Consuming alcoholic beverages, osteoporosis status (Yes or No), ALT, AST, serum creatinine, and total calcium levels. Further studies supported the association link between elevated BMD and AIP. Furthermore, compared to men, females had a higher positive connection between AIP and BMD. In general, there was a curve in the reverse L-shape seen, with a point of change around 0.877, indicating a relationship between AIP and TF BMD. Moreover, a curve exhibiting an L-formed pattern, with a point of inflection at around 0.702, was seen between AIP and FN BMD. In addition, a J-shaped curve was seen, with a point of inflection at 0.092, which demonstrates the association between AIP and LS BMD.

**Conclusion:**

The AIP and TF BMD curves resemble inverted L shapes, as do the AIP and FN BMD curves. The relationship between AIP and LS BMD was further demonstrated by a J-shaped curve. The results indicate a possible association between AIP and bone mineral density, which should be explored in more detail.

**Supplementary Information:**

The online version contains supplementary material available at 10.1186/s12944-024-02180-3.

## Introduction

As bone mineral density (BMD) decreases, fractures increase, particularly in older adults [1]. The rising incidence of osteoporosis and the consequent susceptibility to bone fractures has become a pervasive public health issue due to the growing aging of the global population [2]. In the contemporary healthcare environment, osteoporosis prevention represents a significant challenge [3]. Osteoporosis is a multifaceted long-term condition that is influenced by both genetic and environmental factors. It displays considerable variation, and the current conventional diagnostic approach is insufficient in accurately identifying all individuals who are prone to osteoporotic fractures and providing treatment recommendations [4, 5]. As a result, there is a growing focus on identifying new risk factors or biomarkers for osteoporosis to assess the likelihood of developing the condition, intending to discover new methods of prevention.

Multiple studies have demonstrated a significant relationship between Dyslipidemia and Osteoporosis [6, 7]. Dyslipidemia is a medical condition characterized by high levels of triglycerides and their associated lipoproteins in the bloodstream [8, 9]. Nevertheless, the majority of recent studies have concentrated on examining the association between a solitary variable and BMD [10, 11], and even the opposite results [12–14]. As a result, multiple clinical studies attempt to identify an osteoporosis risk marker that accurately predicts dyslipidemia [15].

The Atherogenic index of plasma (AIP) was originally developed as an innovative biomarker for plasma atherosclerosis, to predict cardiovascular disease [16]. Investigations indicate separate connections between the reduction of BMD and cardiovascular disease [17, 18]. Nevertheless, the relationship between AIP and BMD is still uncertain. In this sense, logarithmic functions are used to convert triglyceride (TG) to high-density lipoprotein cholesterol (HDL-C) [19]. AIP is a measure that considers both TG and HDL-C levels. As well as showing the ratio between TG and HDL-C, it shows lipoprotein particle size as well. With this measurement, dyslipidemia’s specificity and pathogenicity can be ascertained more accurately than by simply measuring TG and HDL-C alone [20]. An investigation of the relationship between AIP and BMD is beneficial for early detection of BMD decline, which is crucial in preventing osteoporosis. Nonetheless, there hasn’t been much research done aimed at the interaction between the AIP and BMD. We conducted a study to investigate the relationship between AIP and BMD, aiming to identify an accessible biological marker for monitoring osteoporosis.

## Materials and methods

### Study sample and data source

Based on the 2007–2018 NHANES, the survey utilized a cross-sectional design. It involves collecting data from a sample of persons who are not in institutions, chosen to reflect a larger population using a specific research design that includes multistage, cross-sectional, subgroup stratified, and probability sampling. Every 2 years, a survey is conducted [21].NCHS Ethics Review Committee approved the NHANES research proposal. Every single participant in the research study supplied a written agreement after being fully informed. Check out at www.cdc.gov/nchs/nhanes/irba98.htm for a more comprehensive overview. The data were analyzed throughout the period from April 1 to April 30, 2024. Detailed information on NCHS IRB/ERB Protocol Number can be found in Supplementary Material.

**Fig. 1:**
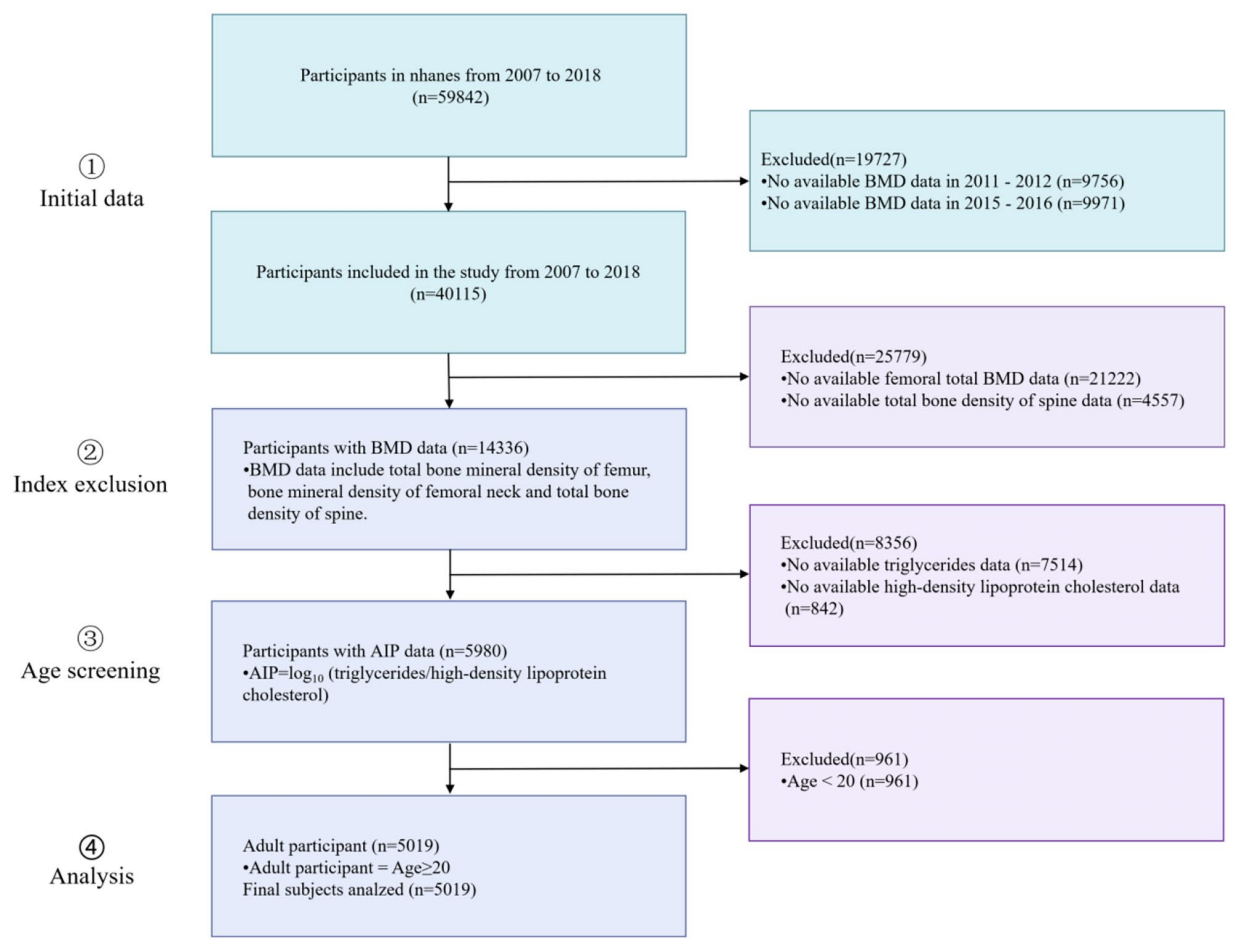
Flow chart of participants selection from the NHANES 2007–2018

59,842 individuals were included in the sample for this cross-sectional study during six consecutive periods (2007–2009, 2009–2010, 2011–2012, 2013–2014, 2015–2016, 2017–2018). Due to the absence of BMD data in the 2011–2012 NHANES and 2015–2016 NHANES, we have chosen to exclude the data from these two years from our analysis. The exclusion criteria were patients age < 20 (*n* = 961) or with missing AIP data (*n* = 8,356), BMD data including total femur (TF), femoral neck (FN), Lumbar spine (LS) BMD (*n* = 25,779). As a result of the study, 5,019 participants were included with complete data. (Fig. 1)

### Exposure variable and outcome variables

AIP is a variable indicative of exposure determined by the mathematical formula lg[TG(mg/dL)/HDL-C(mg/dL)]. Based on their AIP quartiles, the subjects were further partitioned into four groups: Q1 (-0.79, 0.09), Q2 (0.09, 0.30), Q3 (0.30, 0.53), and Q4 (0.53, 2.15).

TF, FN, and LS BMD were included as outcome variables. The NHANES website provides additional information.

### Covariables

The following criteria were used to evaluate the Covariables in this study: [1] data related to the characteristics of the population being studied; [2] factors that have been identified in previous research as influencing AIP and BMD; [3] adherence to the STROBE statement guidelines, which suggest that the basic model should show a change of more than 10% when additional variables are introduced [22]. Thus, we incorporated the subsequent covariables that align with the aforementioned guidelines: age, degree of education, income, sex, race, average daily alcohol consumption in the past 12 months, have been told to suffer from osteoporosis/bone fragility (Yes/No), alanine aminotransferase (ALT, U/L), aspartate transaminase (AST, U/L), serum creatinine (SCr, mg/dL), Total calcium (Tc, mg/dL).

The classification of race/ethnicity included the categories Other Hispanic, Mexican, Non-Hispanic Black, American, Non-Hispanic White, and Other. Three education categories were classified: senior high school or below, above high school, and unknown. The quantity of alcohol consumed is contingent upon the mean number of alcoholic beverages ingested by individuals throughout the previous 12-month period. Additional comprehensive information on covariables may be found in Supplementary Table S1.

### Statistical analysis

The study employed appropriate weighting methodology to consider the intricate sample design, ensuring that the results are representative at a national level, as advised by the NHANES Guidelines [23]. The AIP levels were categorized into Q1-Q4. Counts and percentages (%) were used to represent categorical variables, while means and SD or medians were used to describe continuous variables. Discrepancies among continuous variables were examined using weighted linear regression. Categorical variables were analyzed with Chi-square tests.

According to the STROBE statement [24], the present study utilized three models. Model 1 involved a univariate logistic regression analysis. Model 2 was adjusted for sex, race, and age. Model 3 included additional adjustments for age, degree of education, sex, income, race, alcohol consumption, and information on osteoporosis, ALT, AST, SCr, and TC.

The relationship between AIP and BMD was analyzed using three weighted multivariable linear regression models. We employed three different logistic regression models, each with a weighted factor, to assess the relationships between AIP and BMD. Following that, subgroup analysis was conducted to examine potential interactions and account for confounding categorical characteristics. The subgroup analysis using weighted multivariable logistic regression. Results of the different strata can be considered valid if the interaction *P*-value is not statistically significant. The presence of a distinctive population, however, is suggested by a significant interaction *P*-value.

We analyzed the non-linear associations between AIP and BMD using a generalized additive model (GAM) that employed smooth curve fitting(SCF). The significant inflection points between AIP and BMD were calculated using a recursive algorithm upon detecting non-linearity. The two-part logistic regression model was compared with the logistic regression model with a threshold effect analysis.

The statistical analysis was conducted using EmpowerStats (V2.0.0, www.empowerstats.com) and R (V3.4.3, http://www.R-project.org). A two-sided *P*-value < 0.05 was considered statistically significant.

## Results

### Characteristics of the participants

There were 5,019 participants, including 2451 males and 2568 females. The median age (mean ± SD) was 47.98 ± 15.02 years, and the average value (SD) of the AIP was 0.30 ± 0.33. The participants in the Q4, as compared to those in the lower AIP group, exhibited a higher proportion of males, Mexican Americans, and Other Hispanics. They also had lower levels of education, lower PIR, and a higher prevalence of not being informed about their osteoporosis/bone fragility condition. Additionally, they had higher levels of alcohol intake, ALT, AST, SCr, and Tc, all of which were statistically significant (*P* < 0.05). Significantly, individuals with elevated levels of AIP had greater levels of BMD (all *P* < 0.05).(Table 1).

**Table 1:** Weighted characteristics of the study population based on AIP quartiles

^a^ Variable	AIP Quartiles				*P*-value
	Q1	Q2	Q3	Q4	
**Participants**	1255	1254	1255	1255	
**Age, year**	47.60 ± 15.25	48.19 ± 15.43	48.30 ± 15.13	47.86 ± 14.17	0.6308
**Sex, N(%)**					< 0.0001
Male, N(%)	402(32.33)	577(46.24)	678(53.73)	828(66.35)	
Female, N(%)	853(67.67)	677(53.76)	577(46.27)	427(33.65)	
**Race, N(%)**					< 0.0001
Mexican American, N (%)	68(5.44)	98(7.83)	134(10.68)	153(12.21)	
Other Hispanic, N (%)	57(4.52)	68(5.39)	72(5.76)	92(7.33)	
Non-Hispanic White, N (%)	828(65.96)	876(69.87)	824(65.65)	850(67.7)	
Non-Hispanic Black, N (%)	201(16.02)	139(11.07)	117(9.29)	60(4.77)	
Other races, N (%)	101(8.06)	73(5.84)	108(8.61)	100(7.99)	
**Education, N (%)**					< 0.0001
High school degree or below, N (%)	140(11.17)	185(14.77)	241(19.21)	301(24.00)	
High school degree above, N (%)	1115(88.83)	1069(85.23)	1011(80.52)	953(75.90)	
Unknown			3(0.27)	1(0.09)	
**Income (PIR)**	3.18 ± 1.59	3.11 ± 1.60	3.03 ± 1.61	2.83 ± 1.61	< 0.0001
**Has been informed of a history of osteoporosis, N (%)**					0.0002
Yes, N (%)	87(6.94)	75(5.97)	70(5.6)	35(2.8)	
No, N (%)	1166(92.94)	1173(93.52)	1181(94.11)	1214(96.73)	
Unknown, N (%)	2(0.12)	6(0.51)	4(0.29)	6(0.47)	
**Alcohol consumption**	2.75 ± 1.82	4.06 ± 33.54	5.10 ± 44.38	4.64 ± 35.82	0.2909
**ALT[U/L]**	22.87 ± 23.71	22.79 ± 14.96	25.85 ± 15.57	30.96 ± 21.52	< 0.0001
**AST[U/L]**	26.19 ± 24.98	23.67 ± 11.36	24.87 ± 19.57	26.66 ± 13.74	0.0001
**Total calcium[mg/dl]**	9.35 ± 0.32	9.37 ± 0.35	9.36 ± 0.36	9.40 ± 0.33	0.0029
**Serum creatinine[mg/dl]**	0.84 ± 0.31	0.87 ± 0.32	0.88 ± 0.32	0.90 ± 0.33	< 0.0001
**Total femur bone density[g/cm** ^**2**^ **]**	0.93 ± 0.15	0.95 ± 0.16	0.98 ± 0.15	1.01 ± 0.15	< 0.0001
**Femoral neck bone density[g/cm** ^**2**^ **]**	0.80 ± 0.15	0.81 ± 0.15	0.83 ± 0.14	0.86 ± 0.15	< 0.0001
**Lumbar spine bone density[g/cm** ^**2**^ **]**	1.01 ± 0.15	1.01 ± 0.15	1.03 ± 0.14	1.05 ± 0.15	< 0.0001

**Abbreviations**: AIP, Atherogenic index of plasma; BMD, bone mineral density; ALT, alanine transaminase; AST, aspartate transaminase; PIR, family income-to-poverty ratio

^**a**^ The *P* value was calculated by the weighted linear regression model(%) for categorical variables: the *P* value was calculated by the weighted chi-square test

In this study, following the guidelines of the World Health Organization [25], we classified participants into three categories based on T-scores from the lumbar spine, total femur, and femoral neck: osteoporosis (T-score ≤ − 2.5), osteopenia (− 2.5 < T-score ≤ -1), and normal bone density (− 1 < T-score). Specifically, 383 participants were identified with osteoporosis at the lumbar spine (mean AIP: 0.26, median AIP: 0.24), 86 at the total femur (mean AIP: 0.21, median AIP: 0.18), and 162 at the femoral neck (mean AIP: 0.25, median AIP: 0.24). Additionally, 1275 participants were classified with osteopenia at the lumbar spine (mean AIP: 0.30, median AIP: 0.28), 803 at the total femur (mean AIP: 0.25, median AIP: 0.21), and 1407 at the femoral neck (mean AIP: 0.27, median AIP: 0.25). Lastly, 3361 participants were identified with normal bone density at the lumbar spine (mean AIP: 0.33, median AIP: 0.31), 4130 at the total femur (mean AIP: 0.33, median AIP: 0.31), and 3450 at the femoral neck (mean AIP: 0.34, median AIP: 0.32)(Supplementary Table S2).

### Relationship between BMD and AIP

The statistical significance of the trend persisted among the AIP Q1- 4 groups. Participants in the Q2-4 of AIP exhibited progressively higher levels of TF BMD, FN BMD, and LS BMD in contrast to individuals in the bottom quartile (all *P* for Trend < 0.0001). In the fully adjusted model(Model 3), the FN BMD of Q4 was 0.06(0.05–0.07) units more than that of the Q1 group. Furthermore, in model 3, individuals in group Q4 exhibited a 0.05 (0.04–0.06) unit increase in TF BMD compared to Q1. Group Q4 had a statistically significant increase of 0.04 units (0.03–0.06) in LS BMD compared to group Q1. Additional information may be found in Table 2.

**Table 2:** Association of BMD with AIP in different models among all participants

Exposure	^a^ Total femur bone mineral density		
	Model 1[β(95%CI)]	Model 2[β(95%CI)]	Model 3[β(95%CI)]
**AIP(quartile)**			
**Q1**	1(Ref).	1(Ref).	1(Ref).
**Q2**	0.02 (0.01, 0.03)	0.01 (-0.00, 0.02)	0.01 (0.00, 0.02)
**Q3**	0.05 (0.04, 0.06)	0.03 (0.02, 0.04)	0.04 (0.03, 0.05)
**Q4**	0.08 (0.07, 0.09)	0.06 (0.05, 0.07)	0.06 (0.05, 0.07)
***p*** **for trend**	< 0.0001	< 0.0001	< 0.0001
**Exposure**	^**a**^ **Femoral neck bone mineral density**		
	Model 1[β(95%CI)]	Model 2[β(95%CI)]	Model 3[β(95%CI)]
**AIP(quartile)**			
**Q1**	1(Ref).	1(Ref).	1(Ref).
**Q2**	0.01 (-0.00, 0.02)	0.01 (-0.00, 0.02)	0.01 (-0.00, 0.02)
**Q3**	0.03 (0.02, 0.04)	0.03 (0.02, 0.04)	0.03 (0.02, 0.04)
**Q4**	0.05 (0.04, 0.06)	0.05 (0.04, 0.06)	0.05 (0.04, 0.06)
***p*** **for trend**	< 0.0001	< 0.0001	< 0.0001
**Exposure**	^**a**^ **Lumbar spine bone mineral density**		
	Model 1[β(95%CI)]	Model 2[β(95%CI)]	Model 3[β(95%CI)]
**AIP(quartile)**			
**Q1**	1(Ref).	1(Ref).	1(Ref).
**Q2**	-0.0017 (-0.0130, 0.0096)	-0.0014 (-0.0123, 0.0096)	0.0002 (-0.0105, 0.0109)
**Q3**	0.0164 (0.0049, 0.0278)	0.0176 (0.0065, 0.0287)	0.0212 (0.0103, 0.0322)
**Q4**	0.0351 (0.0236, 0.0467)	0.0352 (0.0237, 0.0467)	0.0414 (0.0299, 0.0528)
***p*** **for trend**	< 0.0001	< 0.0001	< 0.0001

**Abbreviations**: AIP, Atherogenic index of plasma; BMD, bone mineral density; ALT, alanine transaminase; AST, aspartate transaminase; PIR, family income-to-poverty ratio; CI, confidence intervals; SD, standard deviation

^**a**^ Model 1 was adjusted for none; Model 2 was adjusted for age, Sex, race; Model 3 was adjusted for age, Sex, race, degree of education, income(PIR), Average alcoholic drinks per day last 12 Mth, ALT, AST, Total calcium, Serum creatinine, Ever been told you have osteoporosis brittle bones

In the subgroup analyses, which were divided based on age, sex, race, degree of education, and Tc levels, it was found that sex (*P* for interaction = 0.0343), age (*P* for interaction = 0.0016), and Tc (*P* for interaction = 0.0066) were the most significant factors that influenced the relationship between AIP and BMD.

The rise in the AIP resulted in a more pronounced increase in the TF BMD among females compared to males 0.09(0.07–0.11)(Table 2). The results of FN BMD 0.08(0.07–0.10) and LS BMD 0.07(0.05–0.08) are consistent with those of TF BMD analysis. The relationship between Tc levels and the TF BMD becomes more pronounced for participants in the lower quartile Q1, 0.11(0.08–0.13) compared to those in the higher quartile Q4, 0.04(0.02–0.06).The results of FN BMD(Q1, 0.10(0.08–0.13) Q4, 0.03(0.01–0.05)) and LS BMD(Q1, 0.08(0.05–0.11) Q4, 0.03(0.00–0.05)) are consistent with those of TF BMD analysis. Table 3 presents the results.

**Table 3:** Subgroup analysis of the associations between AIP and BMD

Subgroup	Total femur BMD [β(95%CI)]	*p* for interaction	Femoral neck BMD [β(95%CI)]	*p* for interaction	Lumbar spine BMD [β(95%CI)]	*p* for interaction
**Sex**		0.0012		< 0.0001		0.0343
Male	0.05 (0.04, 0.07)		0.03 (0.02, 0.05)		0.04 (0.02, 0.06)	
Female	0.09 (0.07, 0.11)		0.08 (0.07, 0.10)		0.07 (0.05, 0.08)	
**Race/ethnicity**		0.5132		0.5162		0.7169
Mexican American	0.06 (0.03,0.10)		0.05 (0.01, 0.09)		0.04 (0.00, 0.09)	
Other Hispanic	0.04 (-0.00,0.09)		0.03 (-0.02, 0.07)		0.03 (-0.02,0.08)	
Non-Hispanic White	0.07 (0.06, 0.09)		0.06 (0.05, 0.07)		0.05 (0.04, 0.07)	
Non-Hispanic Black	0.04 (0.01, 0.08)		0.04 (0.00, 0.07)		0.03 (-0.01, 0.07)	
Other Race	0.06 (0.01, 0.10)		0.04 (0.00, 0.09)		0.03 (-0.02,0.08)	
**Age**		0.0004		0.0450		0.0016
20–34 years old	0.04 (0.01,0.06)		0.03 (0.01,0.06)		0.02(-0.01,0.05)	
35–49 years old	0.06(0.04,0.08)		0.06(0.04,0.07)		0.02(-0.00,0.04)	
50–64 years old	0.10 (0.08,0.12)		0.08 (0.06,0.10)		0.09 (0.07,0.12)	
65–80 years old	0.08(0.05,0.11)		0.05(0.02,0.08)		0.07 (0.04,0.11)	
**Education**		0.7728		0.8508		0.4412
1	0.07 (0.05, 0.10)		0.06 (0.03, 0.08)		0.06 (0.03, 0.09)	
2	0.07 (0.05, 0.08)		0.05 (0.04, 0.07)		0.05 (0.03, 0.06)	
3	-1.11 (-6.42,4.20)		-0.64 (-5.71,4.42)		-0.41 (-5.98,5.17)	
**Total calcium**		0.0008		< 0.0001		0.0066
Q1	0.11 (0.08, 0.13)		0.10 (0.08, 0.13)		0.08 (0.05, 0.11)	
Q2	0.08 (0.06, 0.10)		0.07 (0.05, 0.09)		0.06 (0.04, 0.08)	
Q3	0.06 (0.03, 0.08)		0.03 (0.01, 0.06)		0.04 (0.01, 0.06)	
Q4	0.04 (0.02, 0.06)		0.03 (0.01, 0.05)		0.03 (0.00, 0.05)	

**Abbreviations**: AIP, Atherogenic index of plasma; BMD, bone mineral density; ALT, alanine transaminase; AST, aspartate transaminase; PIR, family income-to-poverty ratio; CI, confidence intervals; SD, standard deviation; TF BMD, Total femur bone mineral density

### Non-linear relationships

In this study, we conducted a Generalised Additive Model (GAM) analysis and employed SCF techniques to identify any non-linear associations between AIP and BMD. The purpose was to validate and reinforce the findings.

**Fig. 2:**
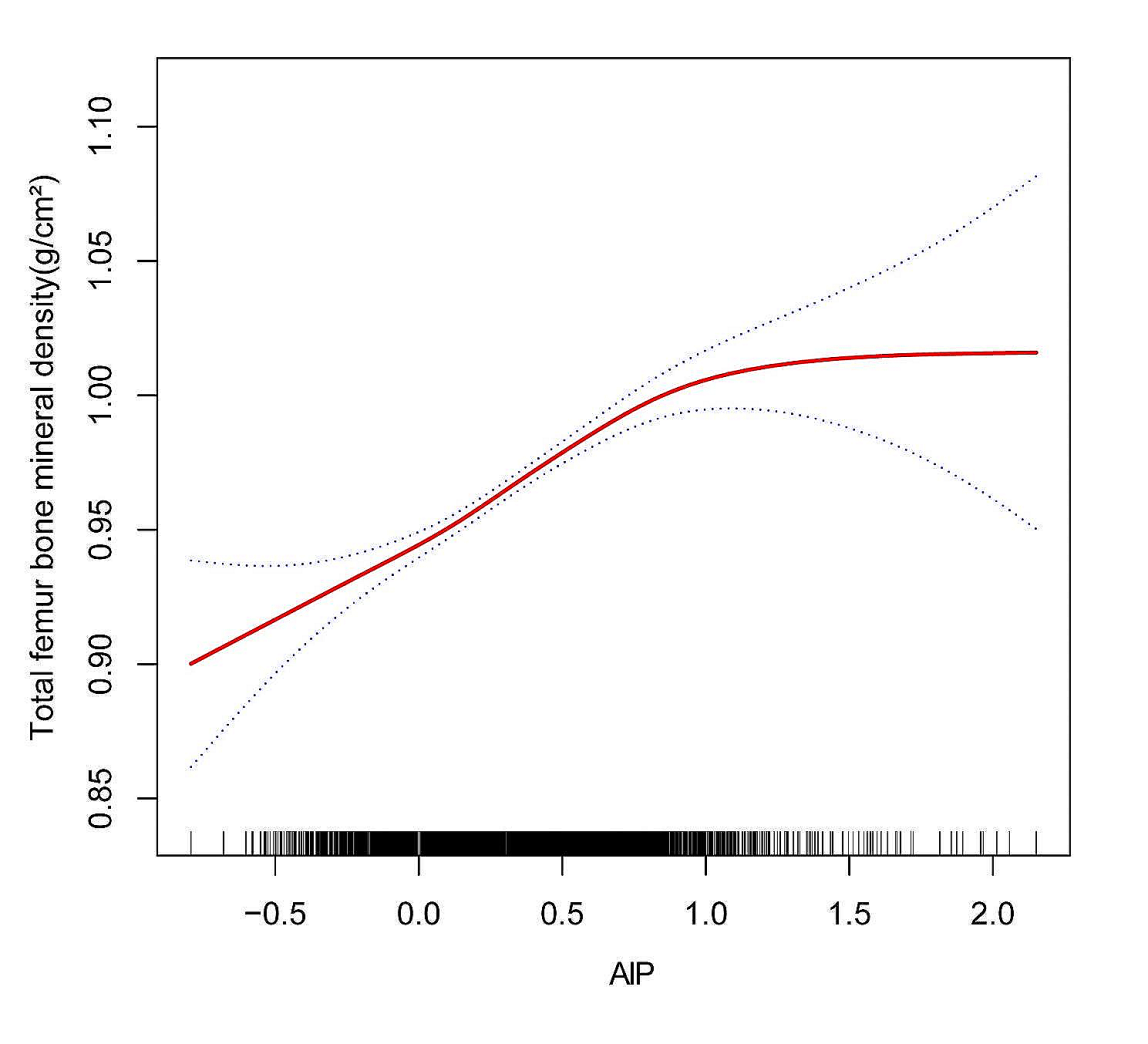
The association between AIP and TF BMD ^a^. Abbreviations: AIP, Atherogenic index of plasma; BMD, bone mineral density; ALT, alanine transaminase; AST, aspartate transaminase; PIR, family income-to-poverty ratio; TF BMD, Total femur bone mineral density. ^**a**^ A solid red line represents the smooth curve fit between variables. Blue bands represent the 95% confidence interval from the fit. Age, Sex, race, degree of education, income(PIR), Average alcoholic drinks per day last 12 Mth, ALT, AST, Total calcium, Serum creatinine, Ever been told you have osteoporosis brittle bones were adjusted. Scf using GAM to evaluate the nonlinear relationship between AIP and TF BMD

According to the fully adjusted model, AIP and TF BMD show a reverse L-formed relationship(Fig. 2). The threshold effect analysis identified an inflection point of 0.877 (Table 4). A strong positive link was discovered between AIP and TF BMD before the turning point, with an odds ratio of 0.098 (0.085, 0.111). Nevertheless, the relationship between AIP and TF BMD lost its statistical significance beyond the inflection point, as indicated by the OR of 0.002 (-0.057, 0.061) with a 95% CI.

**Table 4:** Threshold effect analysis of AIP on TF BMD

Outcome:	Total femur BMD
Model l	
Fitting by the standard linear model	0.089 (0.077, 0.101) < 0.0001
modeII	
Inflection point(K)	0.877
< 0.877	0.098 (0.085,0.111) < 0.0001
> 0.877	0.002(-0.057,0.061)0.9421
Log likelihood ratio	0.003

**Abbreviations**: AIP, Atherogenic index of plasma; BMD, bone mineral density

**Fig. 3:**
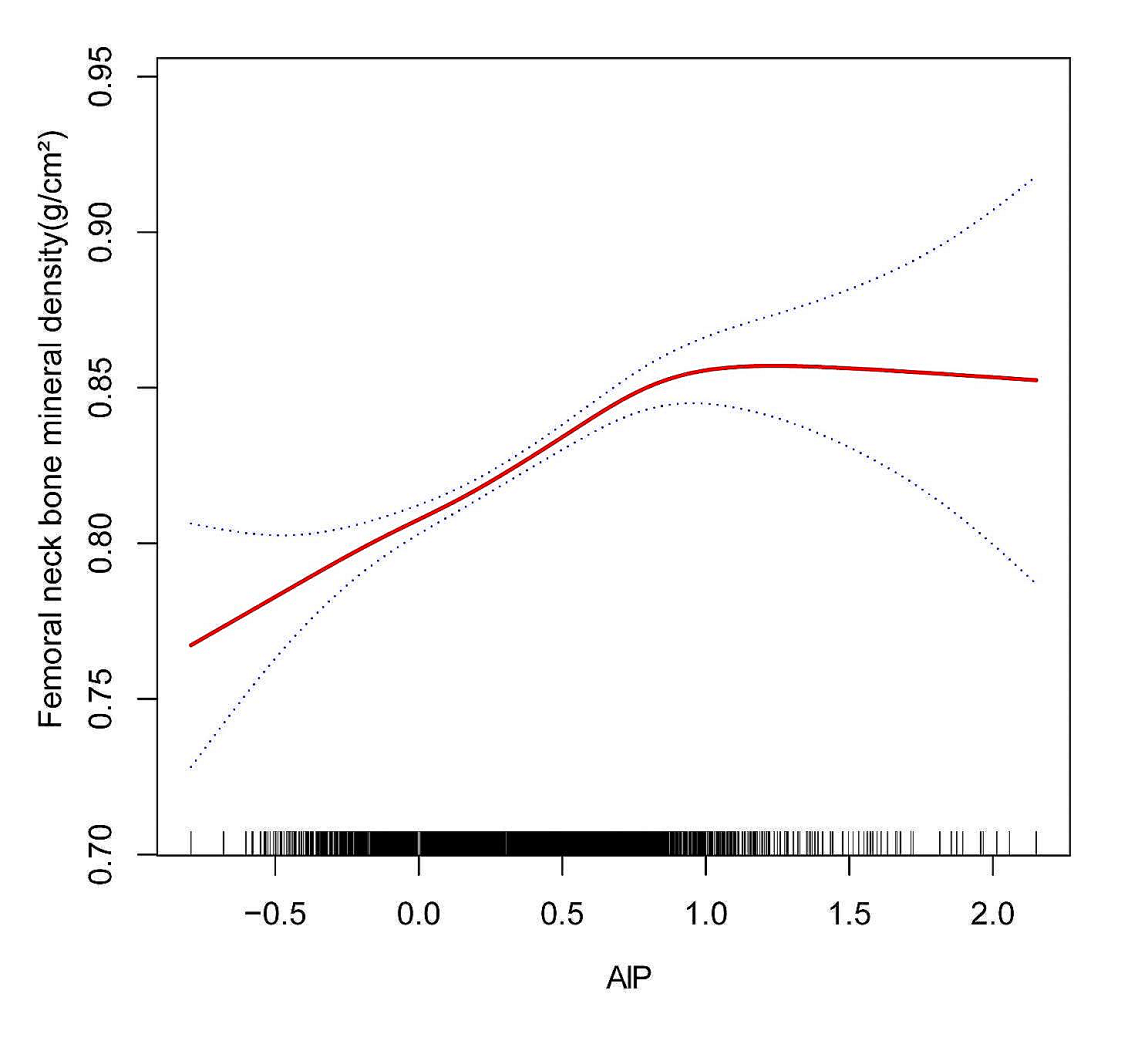
The association between AIP and FN BMD ^a^. Abbreviations: AIP, Atherogenic index of plasma; BMD, bone mineral density; ALT, alanine transaminase; AST, aspartate transaminase; PIR, family income-to-poverty ratio; FN BMD, Femoral neck bone mineral density. ^**a**^ A solid red line represents the smooth curve fit between variables. Blue bands represent the 95% confidence interval from the fit. Age, Sex, race, degree of education, income(PIR), Average alcoholic drinks per day last 12 Mth, ALT, AST, Total calcium, Serum creatinine, Ever been told you have osteoporosis brittle bones were adjusted. Smooth curve fitting using GAM to evaluate the nonlinear relationship between AIP and FN BMD

**Fig. 4:**
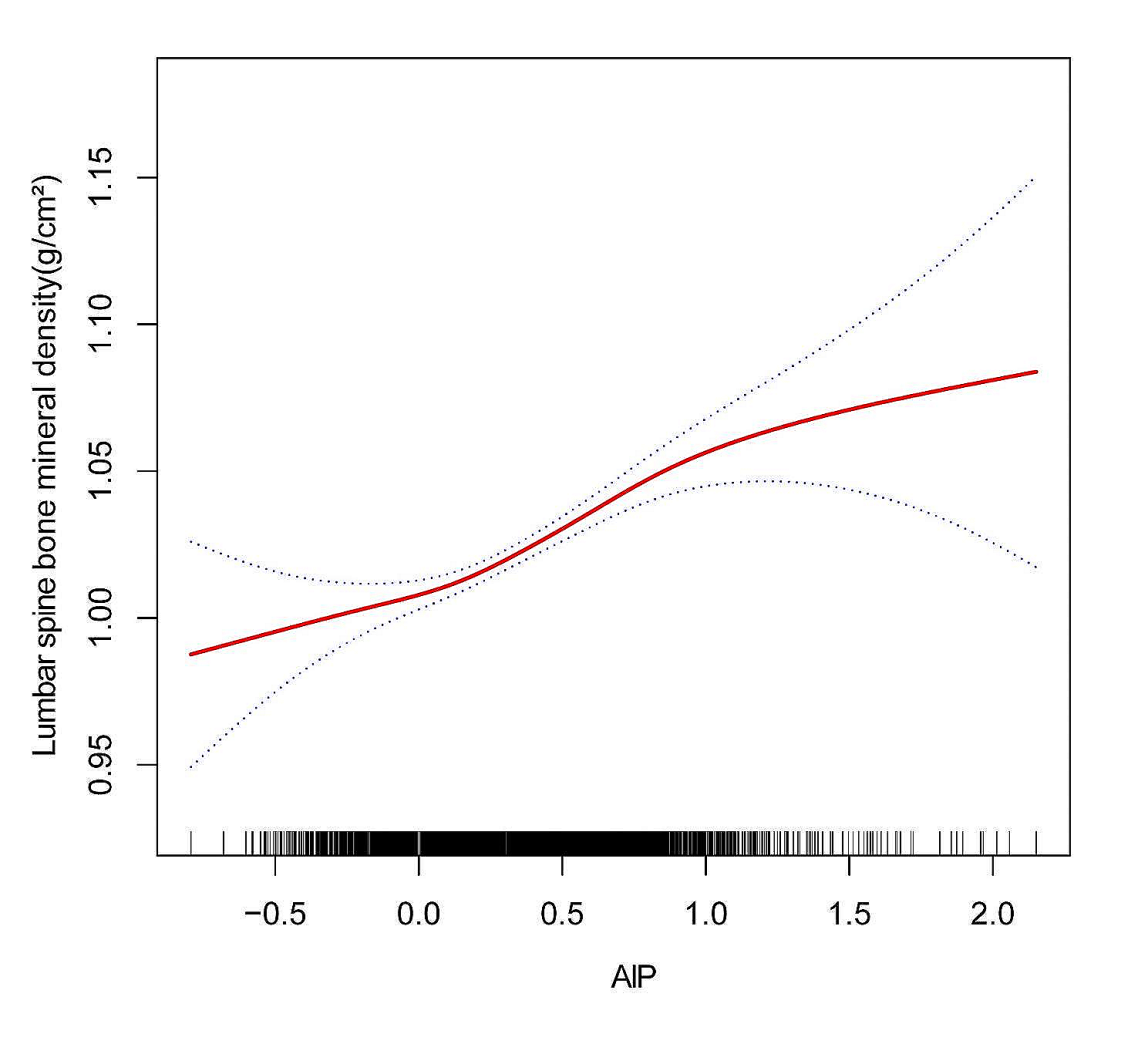
The association between AIP and LS BMD ^a^. Abbreviations: AIP, Atherogenic index of plasma; BMD, bone mineral density; ALT, alanine transaminase; AST, aspartate transaminase; PIR, family income-to-poverty ratio; LS BMD, Lumbar spine bone mineral density. ^**a**^ A solid red line represents the smooth curve fit between variables. Blue bands represent the 95% confidence interval from the fit. Age, Sex, race, degree of education, income(PIR), Average alcoholic drinks per day last 12 Mth, ALT, AST, Total calcium, Serum creatinine, Ever been told you have osteoporosis brittle bones were adjusted. Smooth curve fitting using GAM to evaluate the nonlinear relationship between AIP and LS BMD

In addition, an inverse L-formed relationship was discovered between AIP and FN BMD, with a turning point of 0.702 as determined by threshold effect analysis (Fig. 3; Table 5). When the AIP was < 0.702, the increase in AIP was significantly correlated with the increase in FN BMD (0.077(0.064, 0.090)). However, there was no statistically significant association between AIP and FN BMD when AIP > 0.702 (0.008, (-0.031, 0.046)). Further, a threshold effect analysis revealed a 0.092 inflection point between AIP and LS BMD, revealing a nonlinear and J-shaped relationship between the two (Fig. 4; Table 6). AIP > 0.092 indicated a significant relationship between higher AIP with LS BMD (0.065(0.049, 0.081)). However, there was no statistically significant association between AIP and LS BMD when the AIP value was less than 0.092 (0.014(-0.025, 0.053)). AIP and BMD showed non-linear associations even in the subgroup analysis stratified by age, sex, and total calcium levels (Supplementary 1, Fig. S1, Fig. S2, Fig. S3).

**Table 5:** Threshold effect analysis of AIP on FN BMD

Outcome:	Femoral neck BMD
Model l	
Fitting by the standard linear model	0.065 (0.054, 0.076) < 0.0001
modeII	
Inflection point(K)	0.702
< 0.702	0.077(0.064,0.090) < 0.0001
> 0.702	0.008(-0.04,0.046)0.7024
Log likelihood ratio	0.002

**Abbreviations**: AIP, Atherogenic index of plasma; FN BMD, Femoral neck bone mineral density

**Table 6:** Threshold effect analysis of AIP on LS BMD

Outcome:	Lumbar spine BMD
Model l	
Fitting by the standard linear model	0.054 (0.042, 0.066) < 0.0001
modeII	
Inflection point(K)	0.092
< 0.092	0.014(-0.025,0.053)0.4769
> 0.092	0.065(0.049,0.081) < 0.0001
Log likelihood ratio	0.034

**Abbreviations**: AIP, Atherogenic index of plasma; LS BMD, Lumbar spine bone mineral density

Abbreviations: AIP, Atherogenic index of plasma; BMD, bone mineral density

## Discussion

NHANES data from 2007 to 2018 were analyzed comprehensively in this study. Despite other factors that could influence the results, the study found a positive association between AIP and BMD among US adults. Furthermore, our investigation uncovered an association between AIP and an elevated BMD.

Osteoporosis is a metabolic condition that primarily impacts older persons and is a significant contributor to illness and death [26]. A person’s age, gender, nonalcoholic fatty liver disease, and calcium intake may contribute to osteoporosis [27–29]. Furthermore, there was a notable association between obesity and dyslipidemia, and BMD [30–32]. Compared with other indexes(such as the Systemic Immune-Inflammation Index), AIP offers advantages in predicting the relationship of BMD with lipid metabolism. Osteopenia and osteoporosis were found to be associated with low levels of HDL-C, and TG in young women from Northeast India [33]. The relationship between TG, HDL-C, and BMD in Chinese women postmenopause was also found to be nonlinear [34]. In line with these findings, we found a greater association between AIP and BMD in females than in males. It is commonly known that women who have gone through menopause have a less desirable lipid and bone profile than they did before the event and that this time is linked to higher blood pressure, increased insulin resistance, and central obesity [35]. Additionally, SCF revealed that male and female AIP and BMD differ (Supplementary 1). Also, individuals in the lower quartile exhibit a stronger relationship between their Tc levels and BMD than those in the higher quartile. There is some evidence suggesting interactions between calcium metabolism and lipids. Lipids may influence the deposition of calcium in bones, and conversely, serum calcium levels might impact lipid profiles, which are central to AIP calculations [36]. Among those between 50 and 64, age was the most significant factor related to AIP and BMD. Peripheral artery disease was substantially more common in women with poor femoral neck BMD (1.49, (1.16–1.91)) in the Rotterdam analysis, a prospective cohort analysis of people over the age of 55 [37]. More large-scale prospective studies using prospective study populations are required to better understand the relationship between AIP and BMD by age, TC levels, and sex.

Osteoporosis and atherosclerosis have common traditional cardiovascular risk factors and pathophysiological mechanisms. Various investigations have established a connection between lipid profile and bone metabolism, yielding varying outcomes [38]. Chuang et al. [39], examined 3249 Chinese individuals with a mean age of 58 yrs (71% males; 43% females). The TG/HDL-C ratio was found to be associated with BMD following adjustment for confounding variables. Other research, however, refuted this conclusion. A cross-sectional survey of 481 Chinese older adults in different locations found that TG was positively associated with BMD [40]. It is clear, therefore, from our study that AIP and TF BMD have a reverse L-formed relationship, with an inflection point of 0.877. Additionally, an inverted L curve with an inflection point of 0.702 was observed between AIP and FN BMD. In addition, a J-shaped curve with an inflection point of 0.092 was observed between AIP and LS BMD. Our findings suggest a potential association between AIP and bone mineral density, which warrants further investigation. It may also be suggested from these results that early clinical blood lipid management is required to stop BMD from declining during dyslipidemia and may help in reducing the risk of osteoporosis.

### Limitations and strengths

The data analyzed in this study encompassed not just a single variable but also BMD measurements from various regions (TF BMD, FN BMD, LS BMD). Additionally, a stratified analysis was conducted to examine the relationship between AIP and BMD, taking into account several factors. In this study, confounding variables were assessed and statistical accuracy was enhanced by identifying potential confounding variables. To examine nonlinear associations more precisely, we employed SF curves and logistic regression.

Nevertheless, it is imperative to accept several shortcomings in the current investigation. This study is a cross-sectional study, meaning we cannot determine causality. Hence, additional investigations are required to pinpoint the exact relationship between AIP and BMD. It is also possible that unmeasured factors, such as dietary habits and familial predispositions to bone mineral density, may have influenced the results. This is despite our efforts to account for several factors. Diet has a significant impact on circulating TG levels. The blood samples were obtained following a period of abstaining from food, which could have enhanced the findings. However, additional research is required to ascertain the impact of diet on the conclusions of the study.

## Conclusion

It was observed that the AIP and TF BMD curves resembled an inverted L shape, with an inflection point around 0.702. This suggests an association between the two. However, the AIP and FN BMD curves resemble an inverted L shape, with an inflection point around 0.866. Moreover, a J-shaped curve was identified, with an inflection point of 0.092, illustrating the relationship between AIP and LS BMD. Keeping AIP levels within a certain range might help in reducing the risk of osteoporosis. The causal link must, however, be validated and the underlying mechanisms understood.

### Electronic supplementary material

Below is the link to the electronic supplementary material.

Supplementary Material 1

Supplementary Material 2

## Data Availability

Publicly accessible datasets were analyzed in this study. NHANES data are available at http://www.cdc.gov/nchs/nhanes.htm, the official CDC website.

## Abbreviations

BMD: Bone Mineral Density
AIP: Atherogenic Index of Plasma
TG: Triglyceride
HDL-C: High-Density Lipoprotein Cholesterol
NHANES: National Health and Nutrition Survey
ALT: Alanine Aminotransferase
AST: Aspartate Transaminase
SCr: Serum Creatinine
TC: Total Calcium
GAM: Generalized Additive Model
SCF: Smooth Curve Fitting
TF: Total Femur
FN: Femoral Neck
LS: Lumbar Spine
