# Supplementary material for: Non-linear association of atherogenic index of plasma with bone mineral density a cross-sectional study
Source: Lipids Health Dis. 2024 Jun 12;23:181. doi: 10.1186/s12944-024-02180-3 (PMC11167925; doi:10.1186/s12944-024-02180-3)
Supplement: Supplementary file 1 — Supplementary Material 1 [file 12944_2024_2180_MOESM1_ESM.docx]

Supplementary Material

**Supplementary Table S1** Detailed information on covariables

| Covariable | Range (if continues variable) | Grouping for adjustment |
| --- | --- | --- |
| Age | 20-80 years("80" means ≥ 80 years of age) | 20-34 years |
|  |  | 35-49 years |
|  |  | 50-64 years |
|  |  | 65-80+ years |
| Race | NA | Mexican American |
|  |  | Other Hispanic |
|  |  | Non-Hispanic white |
|  |  | Non-Hispanic black |
| Education level | NA | High school degree or below |
|  |  | High school degree above |
|  |  | Unknown |
| Income(PIR) | PIR: 0.00-5.00 ("5.00" means ≥ 5.00) | 1 (PIR: 0.00-4.98) |
|  |  | 2 (PIR: ≥ 5.00) |
|  |  | 3 (PIR: Unknown) |
| ^a^ ALT | 3.00-890.00 U/L | Q1 (3.00-16.00 U/L) |
|  |  | Q2 (17.00-21.00 U/L) |
|  |  | Q3 (22.00-28.00 U/L) |
|  |  | Q4 (29.00-890.00 U/L) |
| ^a^ AST | 7.00-1034.00 U/L | Q1 (7.00-19.00 U/L) |
|  |  | Q2 (20.00-23.00 U/L) |
|  |  | Q3 (24.00-27.00 U/L) |
|  |  | Q4 (28.00-1034.00 U/L) |
| ^a^ Total calcium | 7.50-11.30 mg/dL | Q1 (7.50-9.10 mg/dL) |
|  |  | Q2 (9.20-9.40 mg/dL) |
|  |  | Q3 (9.50-9.60 mg/dL) |
|  |  | Q4 (9.70-11.30 mg/dL) |
| ^a^ Serum creatinine | 0.31-10.98 mg/dL | Q1 (0.31-0.71 mg/dL) |
|  |  | Q2 (0.72-0.82 mg/dL) |
|  |  | Q3 (0.83-0.98 mg/dL) |
|  |  | Q4 (0.99-10.98 mg/dL) |
| Informed of osteoporosis | NA | Yes (patients with osteoporosis diagnosed by doctors) |
|  |  | No |
| Alcohol consumption | NA | 1 (PIR: 1.00-13.00) |
|  |  | 2 (PIR: ≥ 15.00) |
|  |  | 3 (PIR: Unknown) |

**Abbreviations:** ALT, alanine transaminase; AST, aspartate transaminase; PIR, family income-to-poverty ratio.

^a^ ALT, AST, total calcium, and serum creatinine were divided into four groups according to the quartiles of distribution.

**Supplementary Table S2** Average and median of AIP in different groups

| **^a^ Variable** |  | Quantity | AIP mean | AIP median |
| --- | --- | --- | --- | --- |
| Total femur T-score |  |  |  |  |
|  | Normal bone density | 4130 | 0.33 | 0.31 |
|  | Osteopenia | 803 | 0.25 | 0.21 |
|  | Osteoporosis | 86 | 0.21 | 0.18 |
| Femoral neck T-score |  |  |  |  |
|  | Normal bone density | 3450 | 0.34 | 0.32 |
|  | Osteopenia | 1407 | 0.27 | 0.25 |
|  | Osteoporosis | 162 | 0.25 | 0.24 |
| Lumbar spine T-score |  |  |  |  |
|  | Normal bone density | 3361 | 0.33 | 0.31 |
|  | Osteopenia | 1275 | 0.30 | 0.28 |
|  | Osteoporosis | 383 | 0.26 | 0.24 |

**Abbreviation:** AIP, Atherogenic index of plasma

^a^ Normal bone density (T-score > −1); osteopenia (T-score > −2.5 and ≤ −1); osteoporosis (T-score ≤ −2.5) .

**
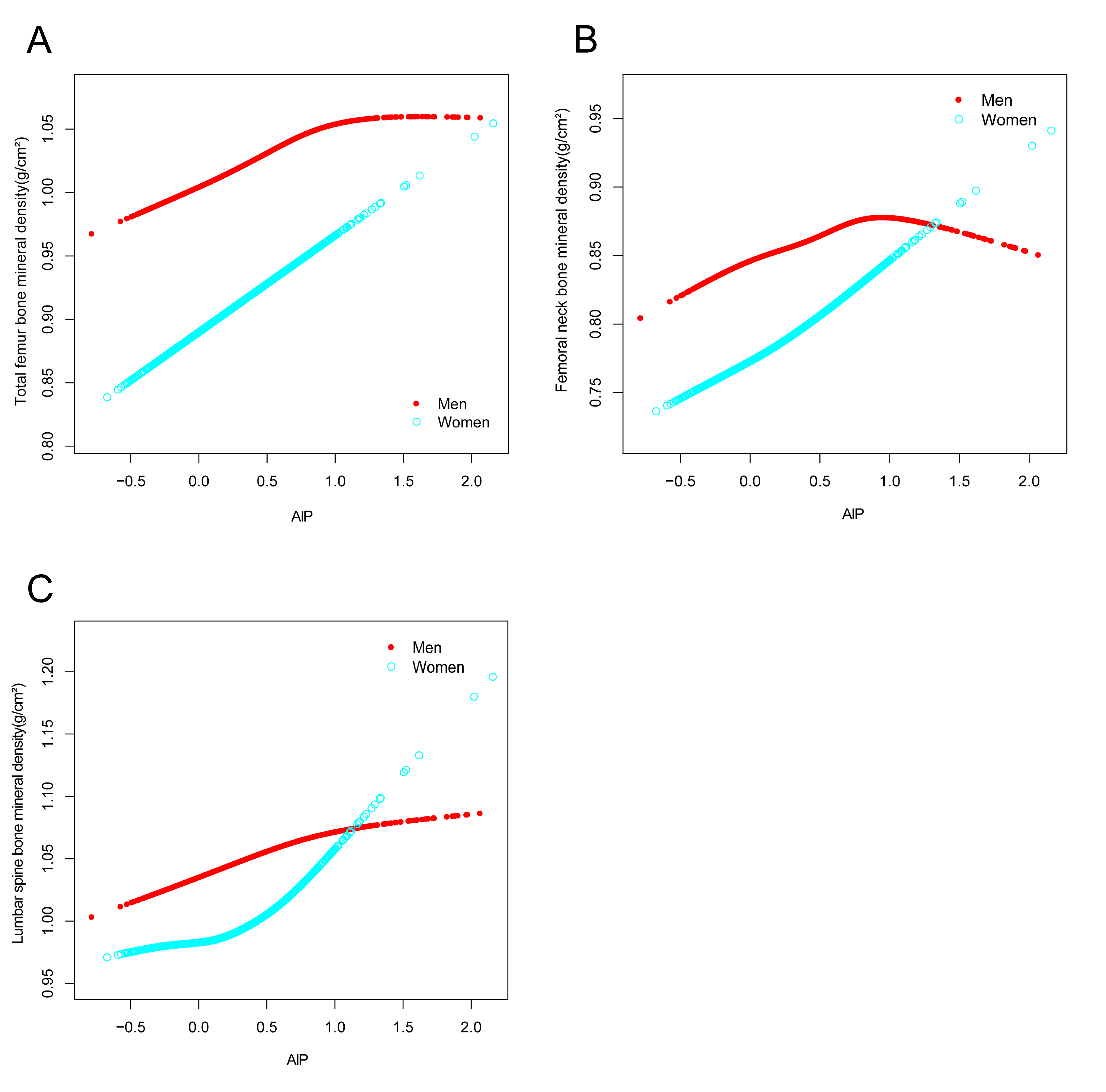
**

**Fig. S1 Association between AIP and BMD stratified by sex.**

A: Association between AIP and TF BMD; B: Association between AIP and FN BMD; C: Association between AIP and LS BMD;

**Abbreviations:** ALT, alanine transaminase; AST, aspartate transaminase; PIR, family income-to-poverty ratio;Tc, Total calcium;Sc, Serum creatinine;

**Adjustment factors:** Adjusted for age, race, education level, income(PIR), Average alcoholic drinks per day last 12 Mth, ALT, AST, TC, Sc, Ever been told you have osteoporosis brittle bones. Each line represents a smooth curve fit between variables.

**
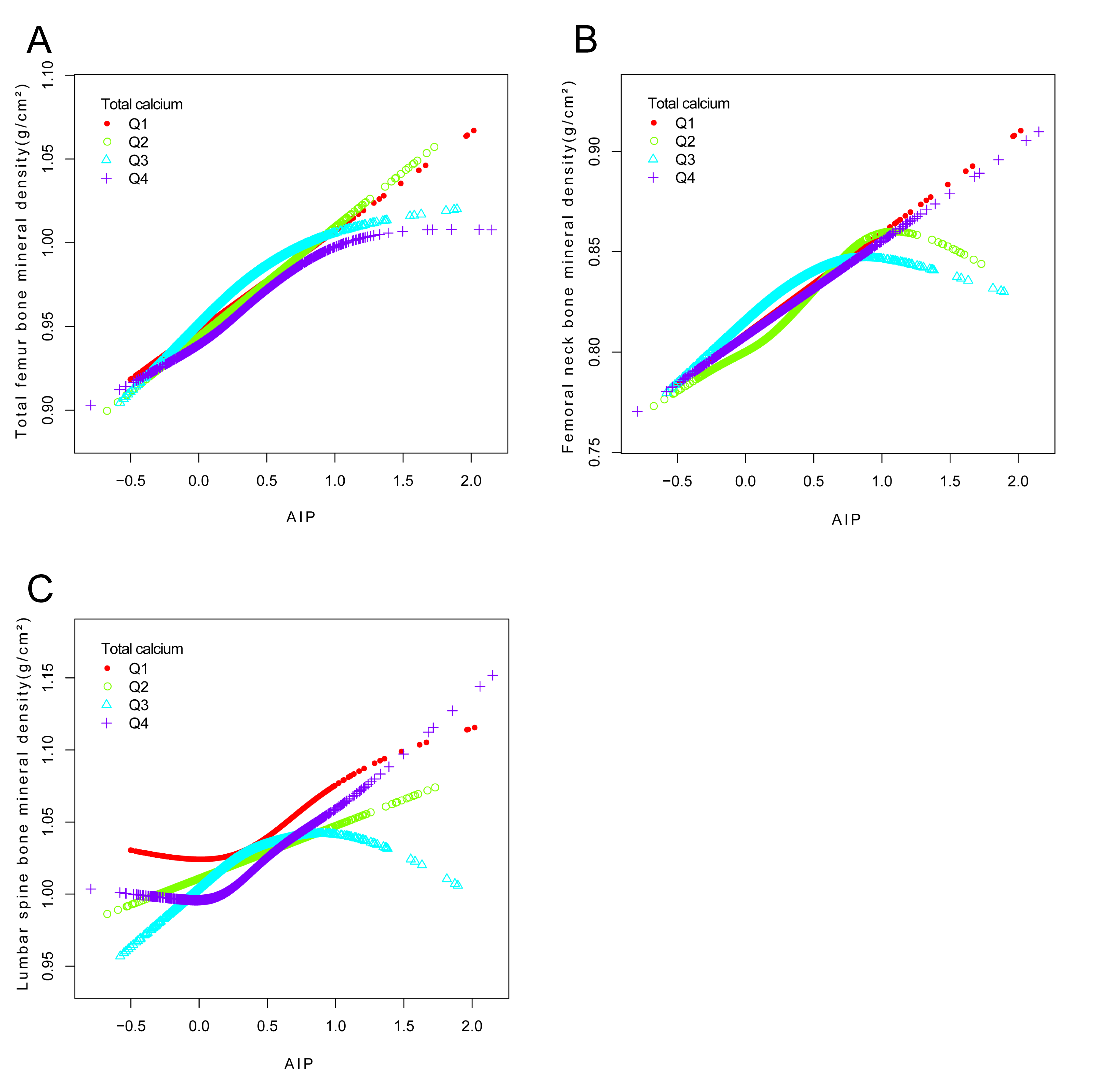
**

**Fig. S2 Association between AIP and BMD stratified by Tc.**

A: Association between AIP and TF BMD; B: Association between AIP and FN BMD; C: Association between AIP and LS BMD;

**Abbreviations:** ALT, alanine transaminase; AST, aspartate transaminase; PIR, family income-to-poverty ratio;Tc, Total calcium;Sc, Serum creatinine;

**Adjustment factors:** Adjusted for age, sex, race, education level, income(PIR), Average alcoholic drinks per day last 12 Mth, ALT, AST, Sc, Ever been told you have osteoporosis brittle bones. Each line represents a smooth curve fit between variables.

**
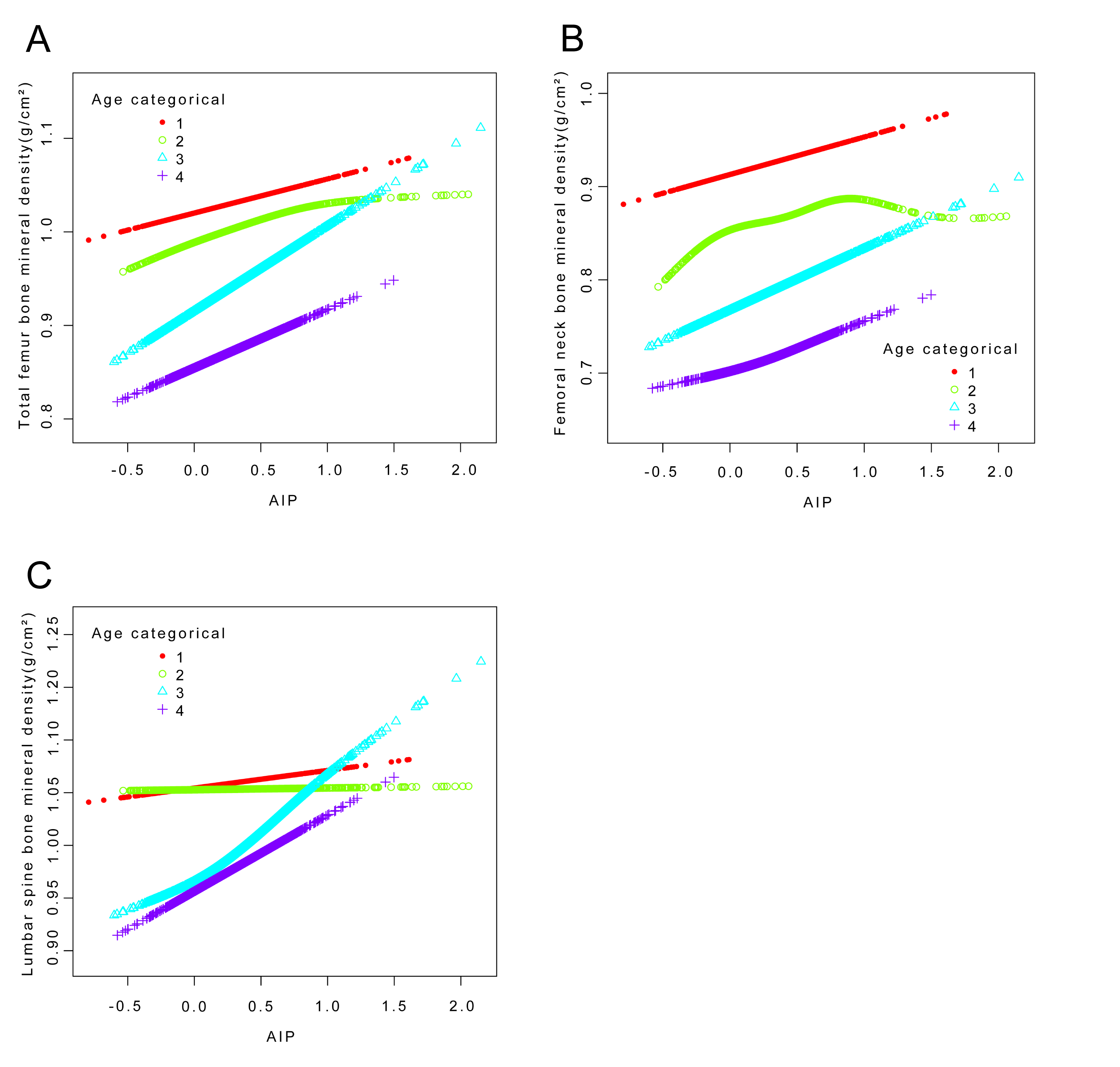
**

**Fig. S3 Association between AIP and BMD stratified by age.**

A: Association between AIP and TF BMD; B: Association between AIP and FN BMD; C: Association between AIP and LS BMD;

**Abbreviation:** ALT, alanine transaminase; AST, aspartate transaminase; PIR, family income-to-poverty ratio;Tc, Total calcium;Sc, Serum creatinine;

**Adjustment factors:** Adjusted for sex, race, education level, income(PIR), Average alcoholic drinks per day last 12 Mth, ALT, AST, TC, Sc, Ever been told you have osteoporosis brittle bones. Age was divided into four groups. Each line represents a smooth curve fit between variables.
